# Lin28 Inhibits the Differentiation from Mouse Embryonic Stem Cells to Glial Lineage Cells through Upregulation of Yap1

**DOI:** 10.1155/2021/6674283

**Published:** 2021-02-22

**Authors:** Juan Luo, Hailin Zou, Liang Deng, Xiang Sun, Ping Yuan, Peng Li

**Affiliations:** ^1^Scientific Research Center, The Seventh Affiliated Hospital of Sun Yat-sen University, Shenzhen 518107, China; ^2^Department of General Surgery, The Seventh Affiliated Hospital of Sun Yat-sen University, Shenzhen 518107, China; ^3^Department of Medical Bioinformatics, Zhongshan School of Medicine, Sun Yat-sen University, Guangzhou 510275, China; ^4^Center for Stem Cell Biology and Tissue Engineering, Key Laboratory for Stem Cells and Tissue Engineering, Ministry of Education, Sun Yat-sen University, Guangzhou 510275, China; ^5^Guangdong Provincial Key Laboratory of Colorectal and Pelvic Floor Disease, The Sixth Affiliated Hospital of Sun Yat-sen University, Guangzhou 510655, China; ^6^Guangdong Institute of Gastroenterology, Guangzhou, Guangdong 510655, China

## Abstract

The RNA-binding protein Lin28 regulates neurogliogenesis in mammals, independently of the let-7 microRNA. However, the detailed regulatory mechanism remains obscured. Here, we established Lin28a or Lin28b overexpression mouse embryonic stem cells (ESCs) and found that these cells expressed similar levels of the core pluripotent factors, such as Oct4 and Sox2, and increased Yap1 but decreased lineage-specific markers compared to the control ESCs. Further differentiation of these ESCs to neuronal and glial lineage cells revealed that Lin28a/b overexpression did not affect the expression of neuronal marker *β*III-tubulin, but dramatically inhibited the glial lineage markers, such as Gfap and Mbp. Interestingly, overexpression of Yap1 in mouse ESCs phenocopied Lin28a/b overexpression ESCs by showing defect in glial cell differentiation. Inhibition of Yap1/Tead-mediated transcription with verteporfin partially rescued the differentiation defect of Lin28a/b overexpression ESCs. Mechanistically, we demonstrated that Lin28 can directly bind to *Yap1* mRNA, and the induction of Yap1 by Lin28a in mESCs is independent of Let7. Taken together, our results unravel a novel Lin28-Yap1 regulatory axis during mESC to glial lineage cell differentiation, which may shed light on glial cell generation *in vitro*.

## 1. Introduction

The RNA-binding proteins Lin28a and Lin28b are homologs originally identified as developmental timing regulators in C. elegans [1, 2]. They are subsequently found to function in a wide spectrum of biological processes, developments, and diseases in mammals, including embryonic stem cell self-renewal, somatic cell reprogramming, metabolism, organismal growth, and tumorigenesis [3–7]. Lin28a/b inhibits the maturation of let-7 family members, the important players in multiple diseases and cancers via their cold-shock domain (CSD), and a pair of CCHC-type zinc finger motifs [8–10]. In addition, Lin28 directly binds active promoters and recruits Tet1 to regulate its target gene expression, demonstrating its dual binding affinity to DNA and RNA in diverse biological processes [11].

Lin28 is widely expressed in a number of tissues from embryo to adult, particularly in the nervous system [6, 12]. *In vivo* study showed that Lin28a knockout leads to reduced neural progenitor cell proliferation and a small brain in mouse, whereas knockout of both Lin28a alleles and one Lin28b allele displays similar but more severe phenotypes than the control, demonstrating the redundant and critical roles of Lin28a/b in the nervous system development [13]. In addition, *in vitro* study revealed that constitutive expression of Lin28a/b results in the promotion of neurogenesis but the block of gliogenesis, independently of the let-7 microRNA [14, 15]. However, what are the downstream targets of Lin28 and how do they mediate Lin28 functions during neurogliogenesis remain largely unknown.

Pluripotent stem cells (PSCs), such as induced pluripotent stem cells (iPSCs) and embryonic stem cells (ESCs), can propagate *in vitro* and differentiate into all adult cells. They therefore provide useful materials to study cell differentiation and hold great promise for disease remodeling, drug discovery, and regenerative medicine [16–20]. To explore the regulatory mechanism of Lin28 during neurogliogenesis, we constitutively overexpressed Lin28a and Lin28b, respectively, in mouse ESCs and then directly differentiated them to neurons and glias *in vitro*. Overexpression of Lin28 strikingly inhibited the expression of glial lineage markers, like Gfap and Mbp, but did not affect the expression of neuronal marker *β*III-tubulin. Interestingly, constitutive overexpression of Yap1 in mESCs showed a similar effect to Lin28a/b OE ESCs during *in vitro* neurogliogenesis, while inhibition of Yap1/Tead-mediated transcriptional output partially rescued these phenotypes. Furthermore, RNA immunoprecipitation and qPCR assays demonstrated Lin28 can directly bind to *Yap1* mRNA, and the induction of Yap1 by Lin28a in mESCs is independent of Let7. Our study reveals a novel Lin28-Yap1 regulatory axis in mESC to glial lineage cell differentiation *in vitro*.

## 2. Materials and Methods

### 2.1. ESC Culture and Differentiation

The mouse embryonic stem cell line used in this study was isolated from wild type C57/BL6 mouse as previously described [21, 22]. Typically, E3.5 embryos at the blastocyst stage were flushed out from the uterus and cultured on mitomycin-C treated mouse embryonic fibroblasts in a 96-well plate with N2B27 medium with 2i (0.4 *μ*M PD0325901 and 3 *μ*M CHIR99021) and LIF (1000 U/ml). The ICM (inner cell mass) outgrowths were treated with 0.05% Trypsin and passaged on a 24-well plate until stable ESC lines were obtained. The ESCs were maintained on feeders under the normal ES medium (DMEM supplemented with 15% FBS, 0.1 mM nonessential amino acids, 0.1 mM 2-mercaptoethanol, 2 mM Glutamine, 100 U/ml penicillin/streptomycin, and 1000 U/ml LIF). To obtain feeder-free ESCs, the ESCs were grown on a 0.1% gelatin-coated dish in 2i + LIF medium. For cell differentiation to neuronal and glial lineage cells, we integrated the differentiation protocols previously described [23, 24]. First, the mouse ESCs were trypsinized to single cells and then replaced at 1 × 10^4^ cells per well of an ultralow adhesion 96-well plate to quickly aggregate and form uniformly sized embryoid bodies in KSR medium (high glucose DMEM supplemented with 15% knockout serum replacement, 0.1 mM nonessential amino acids, 0.1 mM 2-mercaptoethanol, 2 mM Glutamine, 100 U/ml penicillin/streptomycin, and 10 *μ*M SB431542). After 5 days of suspension culture, the embryoid bodies were subjected to adhesion culture in N2 medium (DMEM/F12 supplemented with N2) for 10 days using a 6-well plate coated with 25 ng/ml human fibronectin.

### 2.2. DNA Constructs and Lentivirus Production and Infection

The lentiviral expression constructs pUbi-MCS-3xFlag (GV358), subcloned with mouse Lin28a/b or mouse Yap1, were purchased from the GeneChem company https://www.genechem.com.cn/. For lentiviral production and infection, lentiviral plasmid (1.2 *μ*g), including the overexpressing plasmids or shRNA plasmid (pLKO.1-Puro), together with 0.8 *μ*g of packaging plasmids pSPAX2 (Addgene #12260) and 0.5 *μ*g of envelope expressing plasmid (Addgene #12259) were transiently cotransfected into the 293 T cells using the Lipofectamine 2000 reagent according to the manufacturer's instructions; 48 hours after transfection, the lentivirus supernatant was collected and filtered with 0.45 *μ*m membrane filters (Millipore). mESCs were infected in the presence of 5 *μ*g/mL polybrene and selected with 1 *μ*g/mL puromycin for 72 hours, the oligo sequences of mouse Lin28a shRNA were listed in Supplementary Table 1.

### 2.3. Reverse Transcription and Quantitative Real-Time PCR

Reverse transcription and QRT-PCR assays were conducted following the previously described [22]. Total RNAs of mESCs were extracted using TRIzol reagent (TaKaRa) following the manufacturer's instructions. 1 *μ*g of total RNA was used as templates to perform reverse transcription with the PrimeScript RT reagent Kit (TaKaRa) according to the instructions. Real-time PCR analysis was performed using the Bio-Rad machine with the SYBR Premix Ex Taq (TaKaRa). The generated threshold cycle (CT) value for each transcript was normalized against the CT value of an internal control, like *β*-Actin, and subsequently normalized against the CT value of corresponding transcripts of the control sample. The oligo sequences of RT primers were listed in Supplementary Table 1.

### 2.4. Western Blot Analysis

mESCs or ESC-derived cells on day 5, day 10, and day15 were lysed using the protein lysis buffer (50 mM Tris-HCl, pH 7.5, 100 mM NaCl, 1% tritone X-100, 0.1 mM EDTA, 0.5 mM MgCl2, inhibitors of proteases and phosphatases). Then, we followed the methods previously described [22]. In brief, protein samples were separated by SDS-polyacrylamide gel electrophoresis and transferred to PVDF membranes (Millipore). The membranes were blocked with 5% nonfat dry milk (BD Company) in TBST+0.1% Tween-20 and incubated with primary antibody in TBST+0.1% Tween-20 overnight at 4°C. The primary/secondary antibodies and dilutions used were listed in Supplementary Table 2.

### 2.5. Immunofluorescence Stain

Immunofluorescence stain of ESC differentiated cells was performed as previously describe [21, 25]. Briefly, cells were fixed in 4% paraformaldehyde in phosphate-buffered saline (PBS) at room temperature (RT) for 30 minutes, followed by blocking with 1% BSA in PBS for 1 hour and then primary antibody overnight at 4 degrees. The primary antibodies and dilutions used were listed in Supplementary Table 2. After washing with PBS for 3 times on the second day, the samples were incubated with the appropriate secondary antibodies, conjugated with Alexa Fluor 594 (Molecular Probes) in PBS for 1 hour at room temperature. Cells were then counterstained with 4,6-diamidino-2-phenylindole dilactate (DAKO) for 15 min at RT following the method as described [22]. Images were captured using a Carl Zeiss confocal microscope (LSM 800).

### 2.6. RNA Immunoprecipitation (RIP) Assay

The EZ-Magna RIP™ RNA Binding Protein Immunoprecipitation Kit (No.17-701; Millipore) was used to perform the RIP assay. In brief, around 2 × 107 control and Lin28a-flag overexpressed mESCs were lysed respectively, with RIP lysis buffer provided in the kit. Anti-Flag M2 magnetic beads (No.8223; Sigma) were incubated with lysates, and the Lin28a-flag-RNA complexes were precipitated. The complexes were washed and treated with proteinase K. RNA was extracted using the phenol/chloroform method, and the retrieved RNA was subjected to quantitative real-time RT-PCR with gene-specific primers listed in Supplementary Table 1.

### 2.7. Data Analysis

Statistical significance was determined by the unpaired Student's *t*-test. The *P* value is indicated by asterisks in the Figures (*P* < 0.05 [^∗^] and *P* < 0.01 [^∗∗^]). Differences of *P* < 0.05 and lower were considered statistically significant.

## 3. Results

### 3.1. Lin28a/b Induced Yap1 but Inhibited Lineage-Specific Gene Expression in mESCs

**Figure 1:**
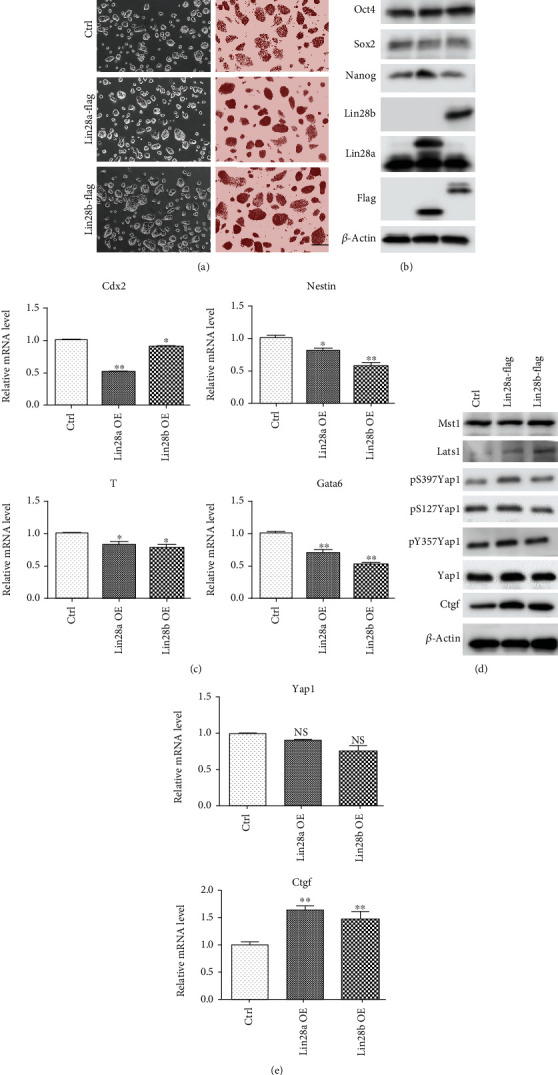
Constitutive expression of Lin28a/b in mouse ESC induced Yap1 expression and reduced lineage-specific gene expression. (a) Phase-contrast microscopy and AP staining of Ctrl and Lin28a/b constitutively expressed (Lin28a/b OE) mouse ESCs grown under 2i + LIF medium. Scale bar, 200 *μ*m. (b) Western blot analyses of total proteins from Ctrl and Lin28a/b OE mouse ESCs using the indicated antibodies. Oct4, Sox2, and Nanog are pluripotent stem cell markers. (c) Quantitative real-time PCR to examine the mRNA level of lineage-specific gene expression in Ctrl and Lin28a/b OE mouse ESCs, trophectoderm gene *Cdx2*, ectoderm gene *Nestin*, mesoderm gene *T*, and endoderm gene *Gata6*. Actin was analyzed as an internal control. The data are shown as the mean ± S.D (*n* = 3). Statistically significant differences were indicated (^∗^, *P* < 0.05 and ^∗∗^, *P* < 0.01). (d) Western blot analyses of total proteins from Ctrl and Lin28a/b OE mouse ESCs using the indicated antibodies. (e) Quantitative real-time PCR to examine the mRNA level of *Yap1* and its downstream target gene *Ctgf* expression in Ctrl and Lin28a/b OE mouse ESCs. The data are shown as the mean ± S.D (*n* = 3).

To investigate the underlying regulatory mechanisms of Lin28 during mESC to neuronal and glial differentiation, Lin28a and 28b stably expressed mESC lines were established respectively, by infecting ESCs with corresponding lentiviruses. Both Lin28a and 28b overexpression (Lin28a/b-Flag OE) cells maintained typical dome-shaped colony morphology and expressed a high level of mESC surface protein alkaline phosphatase like the control ESCs (Figure 1(a)). Consistently, the overall protein level of ESC core pluripotent factors, such as Oct4, Sox2, and Nanog, was not significantly altered upon Lin28a/b overexpression (Figure 1(b)). However, the expression of lineage-specific genes, including *Cdx2*, *Nestin*, *T*, and *Gata6*, was dramatically decreased in Lin28a/b OE mESCs (Figure 1(c)). Previous network-based analyses suggested that Lin28a may be a regulatory factor of the Hippo signaling pathway [26]. Hence, we further examined the expression of key Hippo pathway kinases, such as Mst and Lats, and phosphorylated Yap1 (S127 and 397), which marks Yap1 for cytoplasm localization and degradation through western blot [27]. All these proteins did not show obvious changes upon overexpression of Lin28a/b, but the total protein level of Yap1 and its downstream target Ctgf was dramatically upregulated, suggesting that Lin28a/b promote functional Yap1 protein via other means instead of the canonical Hippo pathway (Figure 1(d)). Further examination of *Yap1* mRNA by qRT-PCR assay revealed that Lin28a/b OE did not affect Yap1 expression at the transcriptional level in mESCs (Figure 1(e)). Additionally, two shRNAs targeting two different regions of the mouse *Lin28a* gene were constructed, and both of them could efficiently reduce Lin28a at both mRNA and protein levels. Consistent with Lin28a/b OE mESCs, knockdown of *Lin28a* significantly reduced the protein level of Yap1 and its downstream target Ctgf, but did not affect *Yap1* transcript level (Figure S1b-c). However, the decrease of *Lin28a* restricted the pluripotent maker expression and quickly induced mESC differentiation, and the qRT-PCR result showed the expression of lineage-specific genes was dramatically upregulated in *Lin28a* stably knockdown mESCs (Figure S1b-c). Taken together, these results demonstrated that Lin28a/b is necessary for maintaining the self-renewal and pluripotency of mES cells, and Lin28 specifically affects Yap1 protein abundance.

### 3.2. Lin28a/b Inhibited the Differentiation of mESCs to Glial Lineage Cells

**Figure 2:**
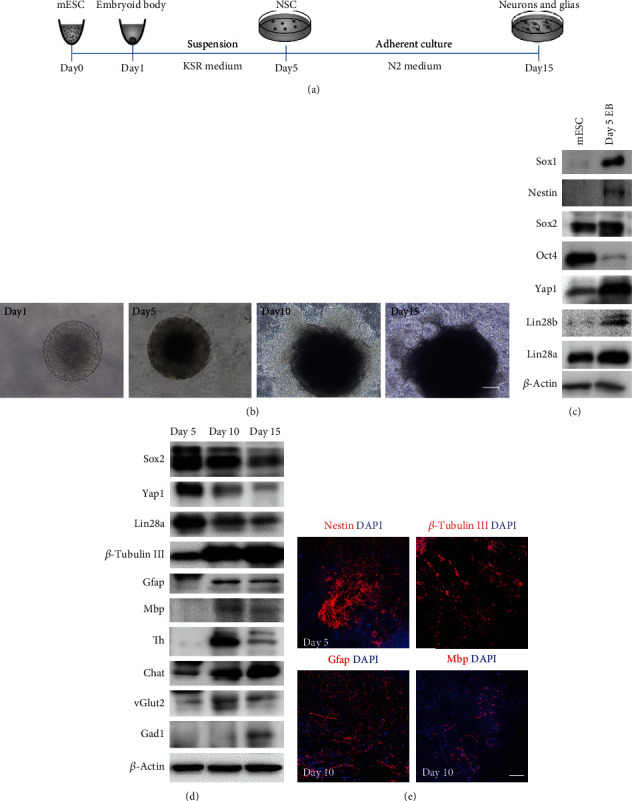
Establishment of the *in vitro* differentiation protocol from mouse ESCs to neuronal and glial lineage cells. (a) A schematic drawing of the direct differentiation assay from mouse ESCs to neuronal and glial lineage cells. ESCs were cultured under feeder-free condition with 2i + LIF medium for 1 day and then disassociated to single cells and quickly aggregated in differentiation medium for one day. After 5 days of suspension culture in KSR medium, the aggregates were subjected to adhesion culture for another 10 or 15 days in N2 medium. (b) Phase-contrast microscopy of mouse ESC differentiated cells on days 1, 5, 10, and 15. Scale bar, 200 *μ*m. (c) Western blot analyses of total proteins from mESC differentiated cells on day 0 and 5 using the indicated antibodies. Mouse ESC pluripotent markers: Oct4 and Sox2, neural stem cell markers: Sox1, Nestin, and Sox2. (d) Western blot analyses of total proteins from mouse ESC differentiated cells on days 5, 10, and 15 using the indicated antibodies. Neuronal marker: *β*-tubulin III, glial markers: Gfap and Mbp, neuronal subtype markers: Th, vGlut2, Gad1, and Chat. (e) Immunofluorescence staining of the neural stem cell marker Nestin on day 5, neuronal marker *β*-tubulin III and glial markers Gfap and Mbp on day 10. Cell nuclear was stained with DAPI. Scale bar, 200 *μ*m.

Accumulating studies have showed that Lin28 regulates ESC proliferation and neurogenesis *in vitro*, but the underlying mechanism is not clear [14, 15]. Here, we utilized previously described protocols to directly differentiate mouse ESCs to neurons and glias *in vitro* [23, 24]. As shown in Figures 2(a) and 2(b), mESCs were trypsinized to single cells and cultured in an ultralow attachment 96-well plate to quickly aggregate and grow uniformly sized embryoid bodies (EBs) in KSR medium. After 5 days of suspension culture, the EBs were subjected to adhesion culture in N2 medium for another 10 days. 5 days of suspension culture in serum-free medium greatly induced cell growth and neural stem cell differentiation. We observed that the neural stem cell markers, including Sox1, Sox2, and Nestin, were dramatically increased, while the pluripotent marker Oct4 was decreased (Figures 2(c) and 2(e)). More interestingly, the protein level of Lin28a/b and Yap1 was upregulated compared to undifferentiated cells (day 0 mESCs), indicating their potential roles in neural lineage induction. Further adherent culture in N2 medium greatly facilitated the neuronal and glial lineage cell differentiation (Figure 2(b)). Western blot and immunofluorescence analyses showed the expression of neuron-specific class III-tubulin (Tubb3), glial fibrillary acidic protein (GFAP), and myelin basic protein (Mbp) was markedly increased after 5 days of adherent culture (Figures 2(d) and 2(e)). Besides, different neuronal subtypes including glutamatergic, GABAergic, dopaminergic, and cholinergic neurons were characterized based on the expression of vesicular glutamate transporter 2 (Vglut2), Gad1, Th, and Chat on day 15 of culture (Figure 2(d)). Taken together, these results manifest the establishment of the mouse ESC to glia cell and functional neuron differentiation system.

**Figure 3:**
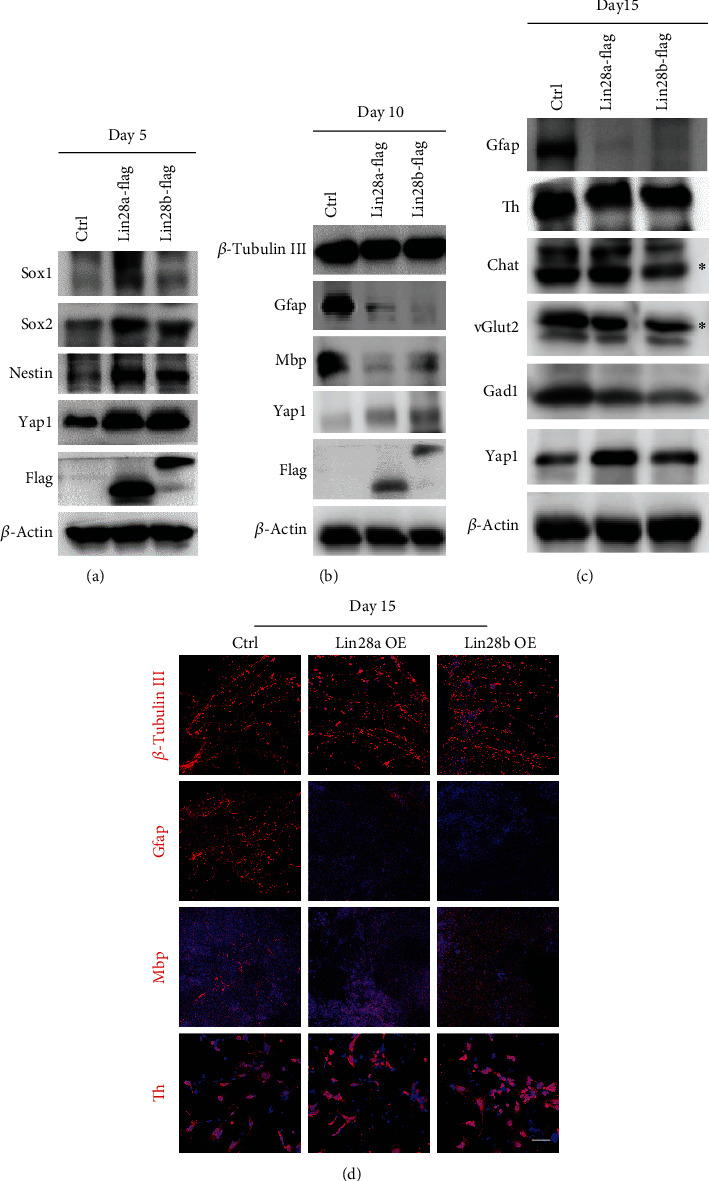
Constitutive overexpression of Lin28a/b inhibited the differentiation of mouse ESCs to glial lineage cells. (a) Western blot analyses of total proteins from Ctrl and Lin28a/b OE mouse ESC differentiated cells on day 5 using the indicated neural stem cell markers: Sox1, Nestin, and Sox2. (b) Western blot analyses of total proteins from Ctrl and Lin28a/b OE mouse ESC differentiated cells on day 10 using the indicated neuronal marker: *β*-tubulin III, glial markers: Gfap and Mbp. (c) Western blot analyses of total proteins from Ctrl and Lin28a/b OE mouse ESC differentiated cells on day 15 using the indicated neuronal subtype markers: Th, vGlut2, Gad1, and Chat. (d) Immunofluorescence staining of the Ctrl and Lin28a/b OE mouse ESC differentiated cells on day 15 using the neuronal marker *β*-tubulin III and glial markers Gfap and Mbp, and neuronal subtype markers Th. Cell nuclear was stained with DAPI. Scale bar, 200 *μ*m.

To assess the role of Lin28a/b in neural lineage differentiation, we conducted neuronal and glial lineage cell differentiation assays as above with Lin28a/b OE ESCs. The expression of Yap1 and neural progenitor markers, Sox1, Sox2, and Nestin was markedly upregulated in Lin28a/b OE cells compared to the control cells at day 5 of differentiation (Figure 3(a)). At day 10 of differentiation, the expression of neuron marker Tubb3 was comparable between control ESCs and Lin28a/b OE ESCs, but the expression of glial cell markers Gfap and Mbp was dramatically lower in Lin28a/b ESCs than the control ESCs (Figure 3(b)). Further extension of the differentiation time to day 15 could not reinstall the expression of Gfap in Lin28a/b cells, while different neuron markers Th, Chat, vGlut2, and Gad1 were expressed in both the control cells and Lin28a/b OE cells (Figure 3(c)). Immunofluorescence analyses also confirmed the expression of the neuronal markers Tubb3 and Th, but not the glial cell marker Gfap and Mbp in Lin28a/b OE ESCs, indicating a repressive role of Lin28a/b in ESC to glial cell differentiation (Figure 3(d)). More interestingly, Yap1 was always higher in Lin28a/b OE cells than the control cells throughout the *in vitro* differentiation procedure, suggesting a positive correlation between Lin28a/b and Yap1 (Figures 3(a)–3(c)).

### 3.3. Yap1 Overexpression mESCs Phenocopied the Glial Cell Lineage Differentiation Defect of Lin28a/b OE ESCs

**Figure 4:**
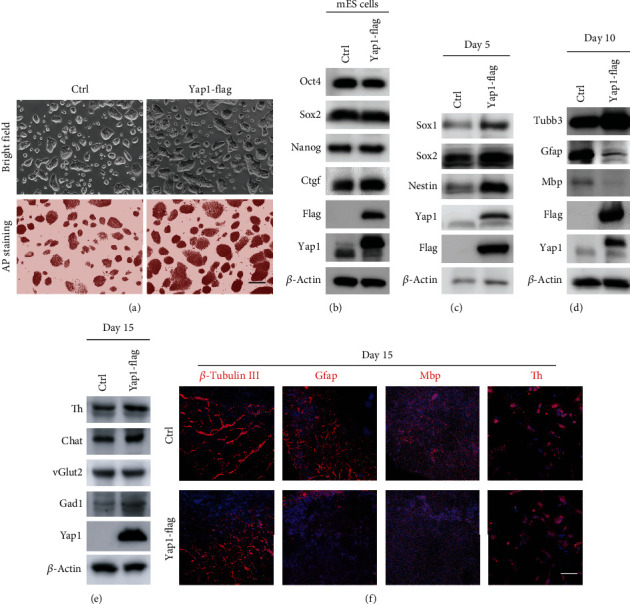
Yap1 overexpression in mESCs phenocopied the glial cell lineage defect differentiated from Lin28a/b OE cells. (a) Phase-contrast microscopy and AP staining of Ctrl and Yap1 constitutively expressed (Yap1 OE) mouse ESCs grown under 2i + LIF medium. Scale bar, 200 *μ*m. (b) Western blot analyses of total proteins from Ctrl and Yap1 OE mouse ESCs using the indicated antibodies. Oct4, Sox2, and Nanog are pluripotent stem cell markers. (c) Western blot analyses of total proteins from Ctrl and Yap1 OE mouse ESC differentiated cells on day 5 using the indicated neural stem cell markers: Sox1, Nestin, and Sox2. (d) Western blot analyses of total proteins from Ctrl and Yap1 OE mouse ESC differentiated cells on day 10 using the indicated neuronal marker: *β*-tubulin III, glial markers: Gfap and Mbp. (e) Western blot analyses of total proteins from Ctrl and Yap1 OE mouse ESC differentiated cells on day 15 using the indicated neuronal subtype markers: Th, vGlut2, Gad1, and Chat. (f) Immunofluorescence staining of the Ctrl and Yap1 OE mouse ESC differentiated cells on day 15 using the neuronal marker *β*-tubulin III, glial markers Gfap and Mbp, and neuronal subtype markers Th. Cell nuclear was stained with DAPI. Scale bar, 200 *μ*m.

To clarify whether upregulation of Yap1 in Lin28a/b OE cells was linked to the inhibition of glial lineage cells during the mouse ESC differentiation, we generated Yap1 overexpression (Yap1-Flag OE) mouse ESCs using lentivirus. These cells also maintained typical dome-shaped colony morphology and expressed a high level of mESC surface protein alkaline phosphatase similar to Lin28a/b OE cells (Figure 4(a)). In addition, Yap1 overexpression did not affect the expression of core pluripotency factors, but dramatically promoted its downstream target Ctgf (Figure 4(b)). Differentiation of Yap1 OE ESCs as above revealed that upregulation of Yap1 indeed promoted the expressions of neural stem cell markers, such as Sox1, Sox2, and Nestin, which is similar to Lin28a/b OE ESCs (Figure 4(c)). Likewise, Yap1 OE ESCs behaved like Lin28a/b OE cells in expressing a low level of glial lineage cell makers Gfap and Mbp, but comparable level of neuronal lineage marker Tubb3 and other functional neuronal markers like Th, Chat, vGlut2, and Gad1 as compared to the control cells upon induced differentiation (Figures 4(d)–4(g)), indicating that Yap1 may be a Lin28a/b effector in controlling of mouse ESC differentiation to neuronal and glial lineage cells.

### 3.4. Inhibition of Yap1-Tead Interaction Using Verteporfin Partially Rescued the Defect of Lin28a/b OE mESC Differentiation to Glial Cell Lineage

**Figure 5:**
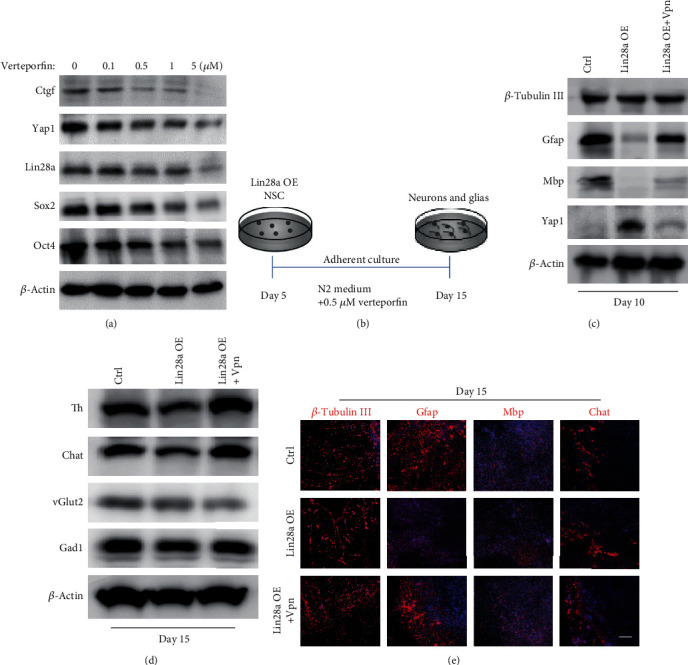
Inhibition of Yap1-Tead interaction using verteporfin partially rescued the glial cell lineage differentiation defect of Lin28a/b OE mouse ESCs. (a) Western blot analyses of total proteins from Ctrl mouse ESCs treated with different concentrations of Yap1-Tead interaction inhibitor (verteporfin) using the indicated antibodies. (b) A schematic drawing of the differentiation assay from neural stem cells (day 5) to neuronal and glial lineage cells (day 15) combining with verteporfin. (c) Western blot analyses of total proteins from Ctrl and Lin28a OE mouse ESC differentiated cells (treated with DMSO and Verteporfin, respectively) on day 10 using the indicated antibodies. (d) Western blot analyses of total proteins from Ctrl and Lin28a OE mouse ESC differentiated cells (treated with DMSO and verteporfin, respectively) on day 15 using the indicated antibodies. (e) Immunofluorescence staining of the Ctrl and Lin28a OE mouse ESC differentiated cells (treated with DMSO and verteporfin, respectively) on day 15 using the neuronal marker *β*-tubulin III, glial markers Gfap and Mbp, and neuronal subtype markers chat. Cell nuclear was stained with DAPI. Scale bar, 200 *μ*m.

Yap1 has been demonstrated to act as a coactivator, and its transcriptional output is mainly dependent on the binding to Tead family transcription factors [28, 29]. To find out whether the upregulation of Yap1-mediated transcriptional output is responsible for the glial lineage differentiation defect of Lin28a/b OE mESC, we introduced the Yap1-Tead interaction inhibitor verteporfin to the *in vitro* differentiation assay of Lin28a OE mESCs. We first treated Lin28a OE ESCs with different concentrations of verteporfin for 24 hours. We found that increasing the inhibitor concentration dramatically reduced Yap1 and Ctgf protein levels, and the expression of Lin28 and core pluripotency factors Oct4 and Sox2 was reduced too (Figure 5(a)). Since 0.5 *μ*M verteporfin noticeably reduced Yap1 function, we next treated the adherent cells differentiated from Lin28a OE mESCs on day 5 at this concentration and then analyzed the expression of neuronal and glial lineage markers by immunoblotting and immunofluorescence assays on day 10 and 15, respectively (Figure 5(b)). We found that inhibition of Yap1-Tead interaction with verteporfin partially rescued the expression of glial lineage markers, such as Gfap and Mbp, but did not affect the expression of Tubb3 and the functional neuronal markers, like Th and Chat in Lin28a OE mESC-differentiated cells (Figures 5(c)–5(e)), indicating that the glial lineage differentiation defect of Lin28a/b OE mESCs was to some extent caused by increased Yap1/Tead-mediated transcriptional output.

### 3.5. Induction of Yap1 by Lin28a/b in Mouse ESCs Was Independent of Let7

**Figure 6:**
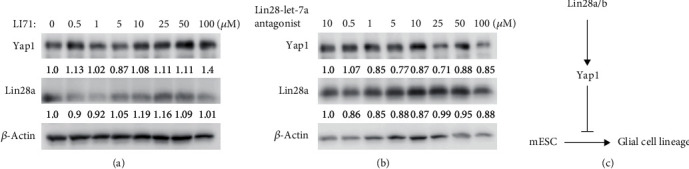
Induction of Yap1 by Lin28a/b in mouse ESCs was independent of Let7 pathway. (a) Western blot analyses of total proteins from Lin28a OE mouse ESCs treated with different concentrations of Lin28-let7 inhibitor (LI71) using the indicated antibodies. (b) Western blot analyses of total proteins from Lin28a OE mouse ESCs treated with different concentrations of Lin28-let7a antagonist using the indicated antibodies. (c) Schematic of the Lin28a/b-Yap1 pathway activity regulating mouse ESC differentiation to glial lineage cells.

Lin28 is well known to be a suppressor of let-7 miRNA biogenesis and let-7b plays a critical role in neural stem cell differentiation [30, 31]. Therefore, we investigated whether Lin28 regulates Yap1 via let-7 miRNA members in mouse ESCs. As Lin28 inhibitor LI71 and Lin28-let-7a antagonist 1 can inhibit Lin28a and let-7 interaction and miRNA processing in ESCs effectively [32, 33], we then treated Lin28a OE mESCs with different concentration of LI71 and Lin28-let-7a antagonist 1 for 24 hours, respectively, for protein analyses. It turned out that increasing the inhibitor concentration did not alter the expression of Yap1 in mouse ESCs (Figures 6(a) and 6(b)), indicating that induction of Yap1 by Lin28a/b overexpression in mouse ESCs was independent of the Let7 pathway. Then, we want to address whether Lin28 can directly bind *Yap1* mRNA to regulate its translation, we performed RNA immunoprecipitation using anti-Flag antibody in Lin28a-Flag overexpressed mESCs. *Tubulin* mRNA was used as a control for nonspecific RNA binding, and *H2a* and *Cyclin B* mRNAs were used as positive controls. We found both of *Yap1* and *Taz* mRNAs exhibited dramatic enrichments in the Lin28a-Flag overexpressed mESCs; these data demonstrated that Lin28 can directly bind to *Yap1* mRNA (Figure S2). Collectively, we reported a novel Lin28a/b-Yap1 regulatory axis in mouse ESC to glial lineage cell differentiation (Figure 6(c)). Lin28a/b may directly bind to Yap1 mRNA to regulate its translation, and the induction of Yap1 by Lin28a in mESCs is independent of canonical Hippo pathway and let-7 family members.

## 4. Discussion

Previous *in vitro* and *in vivo* studies have demonstrated that Lin28a/b plays essential roles during central nervous development, and its functions are independent of the let-7 microRNA [13–15]. However, the downstream targets underlying Lin28 function in neurogliogenesis are not well understood. Here, our studies have identified Yap1 as a crucial downstream effector of Lin28 and demonstrated that Yap1/Tead-mediated transcriptional output is partially responsible for Lin28a/b overexpression induced glial cell differentiation defect from mouse ESCs.

Yap1, a key transcriptional cofactor that is negatively regulated by the Hippo pathway, is essential for the development and size control of multiple organs [34, 35]. In addition, more diverse functions of the Hippo pathway have been recognized, including cell proliferation, differentiation, and migration [12, 36]. Overexpression of Yap1 in mouse ESCs inhibits ESC differentiation and is sufficient to maintain stem cell characteristics. Activation of Yap1 in mouse fibroblasts enhances reprogramming efficiencies of mouse iPSCs. All these evidences, including what we observed in this study and previously described, are consistent with the phenotypes of overexpressing Lin28a/b in mouse ESCs [37–40]. Yap1 is dramatically increased during ESC to neural stem cell differentiation, and Yap1 overexpression promotes the expression of neural progenitor cell markers, including Sox1 and Nestin, indicating its critical role in neural stem cell commitment from ESCs. However, in the late differentiation stage, from day 5-15, Yap1 is gradually decreased. This is consistent with the previous identification of Yap1 as a repressor during neurogliogenesis [38, 41]. The Sox2-Lin28 pathway has been demonstrated to govern the neural progenitor cell proliferation and neurogenesis but repressed the gliogenesis *in vitro* [15, 30]. In our *in vitro* differentiation assays, we observed that the Yap1 expression profile is extremely similar to Sox2 and Lin28, and constitutive overexpression of Yap1 in mouse ESCs restricts the cell differentiation to glial cell lineage, and further inhibition of the Yap1/Tead-mediated transcriptional output partially rescued the differentiation defect of Lin28a/b OE ESCs to glial cell lineage, confirming the critical role of Yap1 during ES to glial cell differentiation.

As the downstream effector of the Hippo pathway, Yap1 is regulated by a highly conserved kinase cascade Mst and Lats [34]. Previous network-based expression analyses have revealed that Lin28 is a possible regulatory nuclear factor of the Hippo pathway in stem cells [26]. Overexpression of Lin28a/b in mouse ESCs does not affect the expression of Yap1 upstream kinases, such as Mst and Lats, and Yap1 phosphorylation levels on the key functional sites, including S127, 397, and Y357, indicating the regulation of Yap1 by Lin28a/b are independent of the canonical Hippo pathway. In our study, inhibition of the Lin28 and Let-7 interaction in mouse ESCs does not alter the Yap1 protein level, suggesting that the induction of Yap1 by Lin28a/b is independent of the Let7 pathway, and some novel mechanisms may be adopted by Lin28 to regulate Yap1 during mESC to glial lineage cell differentiation. Previous studies have shown that Lin28 could either function as a DNA-based regulator or directly affect target mRNA translation and splicing; ChIP-seq data has demonstrated that Lin28a could directly recruit Tet1 and bind to the 5-UTR of *Yap1* in mouse ES cells [11]. However, in our study, we observed that *Yap1* mRNA was not changed, while the Yap1 protein level and its downstream target Ctgf were dramatically induced in the mESC stage and throughout neural lineage differentiation with Lin28-overexpressed cells, suggesting that Lin28a/b may participate in the posttranscriptional regulation of *Yap1* expression. In mouse and human ES cells, several studies have shown that Lin28 regulated the expression of cell cycle-related and pluripotency-associated genes, such as *Cyclin B* and *Oct4* by directly binding to these target mRNAs and enhancing their translation [42–44]. Indeed, our RNA-IP and qPCR assays also validated that Lin28a can directly bind to *Yap1* mRNA, which is similar to the regulation of *H2a* and *Cyclin B* by Lin28a in mouse ESCs. Collectively, our study supports the hypothesis that Lin28a/b directly binds to *Yap1* mRNA and participates in its translation regulation, and further elucidating the regulatory mechanism of Yap1 by Lin28 would be necessary in the future.

## Data Availability

Data can be available on request.

## Supplementary Materials

Supplementary Materials Supplementary Table 1: oligo sequences of primers used in this paper. Supplementary Table 2: antibody list used in this paper. Supplementary Figure S1: (a) phase-contrast microscopy of shNC and Lin28a stable knockdown mouse ESCs grown under 2i + LIF medium. Scale bar, 200 *μ*m. (b) Western blot analyses of total proteins from shNC and Lin28a stable knockdown mouse ESCs using the indicated antibodies. (c) qRT-PCR to examine the mRNA level of Lin28a, Yap1, Ctgf, and lineage-specific gene expression in shNC and Lin28a stable knockdown mouse ESCs. The data are shown as the mean ± S.D (*n* = 3). Statistically significant differences were indicated (^∗^, *P* < 0.05 and ^∗∗^, *P* < 0.01). Supplementary Figure S2: Ctrl and Lin28-Flag overexpressed mouse ES cells were used to do Flag immunoprecipitation (Flag-IP), and RNA samples extracted from IP complexes were reverse-transcripted to generate cDNAs, followed by qPCR using the following gene primers. mRNA levels present in Lin28-Flag overexpressed mouse ES cells relative to control are shown. Each bar represents mean ± S.D (*n* = 3).
