# Supplementary material for: Lin28 Inhibits the Differentiation from Mouse Embryonic Stem Cells to Glial Lineage Cells through Upregulation of Yap1
Source: Stem Cells Int. 2021 Feb 22;2021:6674283. doi: 10.1155/2021/6674283 (PMC7920735; doi:10.1155/2021/6674283)
Supplement: Supplementary Materials — Supplementary Table 1: oligo sequences of primers used in this paper. Supplementary Table 2: antibody list used in this paper. Supplementary Figure S1: (a) phase-contrast microscopy of shNC and Lin28a stable knockdown mouse ESCs grown under 2i + LIF medium. Scale bar, 200 μm. (b) Western blot analyses of total proteins from shNC and Lin28a stable knockdown mouse ESCs using the indicated antibodies. (c) qRT-PCR to examine the mRNA level of Lin28a, Yap1, Ctgf, and lineage-specific gene expression in shNC and Lin28a stable knockdown mouse ESCs. The data are shown as the mean ± S.D (n = 3). Statistically significant differences were indicated (∗, P < 0.05 and ∗∗, P < 0.01). Supplementary Figure S2: Ctrl and Lin28-Flag overexpressed mouse ES cells were used to do Flag immunoprecipitation (Flag-IP), and RNA samples extracted from IP complexes were reverse-transcripted to generate cDNAs, followed by qPCR using the following gene primers. mRNA levels present in Lin28-Flag overexpressed mouse ES cells relative to control are shown. Each bar represents mean ± S.D (n = 3). [file 6674283.f1.zip › Supplementary Table 1.docx]

Supplementary Table 1: List of oligos used in this study

| Target gene | Oligo sequences | Experiment used |
| --- | --- | --- |
| *Actin* | Forward: 5'-ACCAACTGGGACGACATGGAGA-3'  Reverse: 5'-TACGACCAGAGGCATACAGGGAC-3' | QRT-PCR |
| *Cdx2* | Forward: 5'-CCTGCGACAAGGGCTTGTTTAG-3'  Reverse: 5'-TCCCGACTTCCCTTCACCATAC-3' | QRT-PCR |
| *Nestin* | Forward: 5’-AGATCGCTCAGATCCTGGAA-3’  Reverse: 5’-GAGTTCTCAGCCTCCAGCAG-3’ | QRT-PCR |
| *T* | Forward: 5'-CATCGGAACAGCTCTCCAACCTAT-3'  Reverse: 5'-GTGGGCTGGCGTTATGACTCA-3' | QRT-PCR |
| *Gata6* | Forward: 5'-TGCAAGATTGCATCATGACAGA-3'  Reverse: 5'-TGACCTCAGATCAGCCACGTTA-3' | QRT-PCR |
| *Yap1* | Forward: 5’-ACCCTCGTTTTGCCATGAAC-3’  Reverse: 5’-CCTTCTCCATCTGTAACTGC-3’ | QRT-PCR |
| *Ctgf* | Forward: 5’-AGCCTCAAACTCCAAACACC-3’  Reverse: 5’-CAACAGGGATTTGACCAC-3’ | QRT-PCR |
| *Lin28a* | Forward: 5’- GTCTTTGTGCACCAGAGCAA-3’  Reverse: 5’- CTTTGGATCTTCGCTTCTGC-3’ | QRT-PCR |
| shmLin28a-1 | 5’-CCGGCCCAGTAAGAATGCAACTTAACTCGAGTTAAGTTGCATTCTTACTGGGTTTTTT-3’ | Knockdown of mLin28a |
| shmLin28a-2 | 5’-CCGGTAGAAGGCTGTGTGATATTTCCTCGAG-GAAATATCACACAGCCTTCTATTTTTT-3’ | Knockdown of mLin28a |
| *Tubulin* | Forward: 5’-CGTGTTCGGCCAGAGTGGTGC-3’  Reverse: 5’-GGGTGAGGGCATGACGCTGAA-3’ | RIP-qPCR |
| *H2a* | Forward: 5’-GGCGGTGCTGGAGTACCTA-3’  Reverse: 5‘-GATGATGCGCGTCTTCTTG-3’ | RIP-qPCR |
| *Cyclin B* | Forward: 5’-TCCCTCGGTGGGATTCAAGTGC-3’  Reverse: 5’-CAGGAGTGGCGCCTTGGTATGG-3‘ | RIP-qPCR |
| *Yap1* | Forward: 5’-ACCCTCGTTTTGCCATGAAC-3’  Reverse: 5’-CCTTCTCCATCTGTAACTGC-3’ | RIP-qPCR |
| *Taz* | Forward: 5’-TTAGGATGCGTCAAGAGGAG-3’  Reverse: 5’-CTCATATCTGTGCTCATGGC-3’ | RIP-qPCR |
