# Supplementary material for: Lin28 Inhibits the Differentiation from Mouse Embryonic Stem Cells to Glial Lineage Cells through Upregulation of Yap1
Source: Stem Cells Int. 2021 Feb 22;2021:6674283. doi: 10.1155/2021/6674283 (PMC7920735; doi:10.1155/2021/6674283)
Supplement: Supplementary Materials — Supplementary Table 1: oligo sequences of primers used in this paper. Supplementary Table 2: antibody list used in this paper. Supplementary Figure S1: (a) phase-contrast microscopy of shNC and Lin28a stable knockdown mouse ESCs grown under 2i + LIF medium. Scale bar, 200 μm. (b) Western blot analyses of total proteins from shNC and Lin28a stable knockdown mouse ESCs using the indicated antibodies. (c) qRT-PCR to examine the mRNA level of Lin28a, Yap1, Ctgf, and lineage-specific gene expression in shNC and Lin28a stable knockdown mouse ESCs. The data are shown as the mean ± S.D (n = 3). Statistically significant differences were indicated (∗, P < 0.05 and ∗∗, P < 0.01). Supplementary Figure S2: Ctrl and Lin28-Flag overexpressed mouse ES cells were used to do Flag immunoprecipitation (Flag-IP), and RNA samples extracted from IP complexes were reverse-transcripted to generate cDNAs, followed by qPCR using the following gene primers. mRNA levels present in Lin28-Flag overexpressed mouse ES cells relative to control are shown. Each bar represents mean ± S.D (n = 3). [file 6674283.f1.zip › Supplementary Table 2.docx]

**Supplementary Table 2: List of antibodies used in this study**

| **Target Protein** | **Description** | **Company**  **Cat no.** | **Working conc** | **Experiment used** |
| --- | --- | --- | --- | --- |
| Tubb3 | βⅢ-Tubulin Rabbit mAb | ABclonal A17014 | 1:100 | IF |
| Chat | CHAT Rabbit pAb | ABclonal A13244 | 1:100 | IF |
| Nestin | Anti-Nestin rabbit polyclonal antibody | Huabio R1510-20 | 1:100 | IF |
| Th | TH Rabbit pAb | ABclonal A12756 | 1:100 | IF |
| Mbp | Myelin Basic Protein Rabbit pAb | ABclonal A11162 | 1:100 | IF |
| Gfap | GFAP Rabbit pAb | ABclonal A14673 | 1:100 | IF |
| Rabbit IgG | Goat-anti-Rabbit-IgG-H-L-Cross-Adsorbed-Secondary-Antibody-Polyclonal | Thermofisher  A-11072 | 1:2000 | IF |
| Oct4 | Oct-3/4(C-10)a mouse monoclonal antibody | Santa cruz sc-5279 | 1:500 | WB |
| Sox2 | [KO Validated] SOX2 Rabbit pAb | ABclonal A0561 | 1:3000 | WB |
| Nanog | Anti-NANOG rabbit polyclonal antibody | Sangon Biotech D155241 | 1:500 | WB |
| Lin28a | Lin28A-Spectific Rabbit Polyclonal antibody | Proteintech 16177-1-AP | 1:1000 | WB |
| Lin28b | Lin28B-Spectific Rabbit Polyclonal antibody | Proteintech 16178-1-AP | 1:1000 | WB |
| Flag | Mouse anti DDDDK-Tag mAb | ABclonal AE005 | 1:3000 | WB |
| Mst1 | STK4 Rabbit pAb | ABclonal A8043 | 1:1000 | WB |
| LatS1 | LATS1 Rabbit Polyclonal antibody | Proteintech 17049-1-AP | 1:1000 | WB |
| pY357Yap1 | Anti-YAP1（Phospho Y357） | Ab62751 | 1:1000 | WB |
| pS397Yap1 | Phospho-YAP1-S397(D1E7Y） Rabbit pAb | CST #13619 | 1:1000 | WB |
| pS127Yap1 | Phospho-YAP1-S127 Rabbit pAb | CST #4911 | 1:1000 | WB |
| Yap1 | [KO Validated] YAP1 | ABclonal A1002 | 1:3000 | WB |
| Ctgf | CTGF Rabbit Polyclonal antibody | Proteintech 23936-1-AP | 1:1000 | WB |
| Sox1 | Anti-SOX1 rabbit polyclonal antibody | Sangon Biotech D160750 | 1:500 | WB |
| Nestin | Nestin Rabbit Polyclonal antibody | Proteintech 19483-1-AP | 1:500 | WB |
| Tubb3 | βⅢ-Tubulin Rabbit mAb | ABclonal A17913 | 1:500 | WB |
| Gfap | GFAP Rabbit pAb | ABclonal A14673 | 1:500 | WB |
| Mbp | Myelin Basic Protein Rabbit pAb | ABclonal A11162 | 1:500 | WB |
| Th | TH Rabbit pAb | ABclonal A12756 | 1:500 | WB |
| Chat | CHAT Rabbit pAb | ABclonal A13244 | 1:500 | WB |
| vGlut2 | SLC17A6 Rabbit pAb | ABclonal A15177 | 1:500 | WB |
| Gad1 | GAD1 Rabbit pAb | ABclonal A2938 | 1:500 | WB |
| Actin | ACTB Monoclonal Antibody | ABclonal AC004 | 1:5000 | WB |
| Rabbit IgG | HRP Goat Anti-Rabbit IgG（H+L） | ABclonal AS014 | 1:5000 | WB |
| Mouse IgG | HRP Goat Anti-Mouse IgG（H+L） | ABclonal AS003 | 1:5000 | WB |
